# Retraction: Celastrol Inhibits Lipopolysaccharide-Stimulated Rheumatoid Fibroblast-Like Synoviocyte Invasion through Suppression of TLR4/NF-κB-Mediated Matrix Metalloproteinase-9 Expression

**DOI:** 10.1371/journal.pone.0316421

**Published:** 2025-03-12

**Authors:** The PLOS ONE Editors

After this article [1] was published, multiple image concerns were raised about Figs 2, 4, 5 and 7. In summary:

- Multiple blot and gel image panels or parts of panels appear similar to other panels within the article [1] or in other published articles by the same research group. Specifically, similarities were noted between parts of the following:
  - Fig. 2B of [1] and Fig. 5D of [1]
  - Fig. 4B of [1], and Figs 1–2 of [2] (retracted in [3]) and Fig. 2 of [4]
  - Fig. 5A of [1], Fig. 5A in [5], and Fig. 3E in [6] (which was subsequently corrected in [7])
  - Fig. 5B of [1] and Fig. 3D in [4]
  - Fig. 5C of [1], Fig. 5B in [5], and Fig. 3F in [6] (which was subsequently corrected in [7])
  - Fig. 5D of [1], Fig. 2A in [2] (retracted in [3]), and Fig. 5C of [5]
- In Fig. 2E of [1] there appear to be multiple regions of similarity within all microscopy panels. Some panels also appear similar to multiple regions within panels in Fig. 2D of [2] (retracted in [3]).
- In Fig. 5C of [1], bands in multiple lanes of the input panel appear similar to each other.
- In Fig.7 of [1] there appear to be multiple regions of similarity within microscopy panels A and D, which also appear similar to Figs 5A and 5D, respectively, in [2] (retracted in [3]).

The corresponding author noted that several studies were carried out during the same time period and there were issues in experimental data management. They provided underlying image files for the above-mentioned figure panels in [1]. However, editorial evaluation determined that the provided data did not resolve the above image concerns.

In light of the above concerns, which undermine the reliability and integrity of the data and conclusions, the *PLOS ONE* Editors retract this article.

GL did not agree with the retraction. DL, YZ, YQ, HZ, SG, MS, TH, and YL either did not respond directly or could not be reached.

The Control–Migrating cells, Control–Invasive cells, and MMP-9 siRNA–Invasive cells panels in Fig. 2E, the MMP-9 and β–actin panels in Fig. 4B, lane 4 of the IkBα panel in Fig. 5D, and Fig. 7A and 7D are similar to material from [2], published in 2012 by Springer Nature, which are not offered under a CC BY license and are therefore excluded from this article’s [1] license.

The β–actin panel in Fig. 4B and lanes 1–3 in Fig. 5B are similar to material from [4], published in 2012 by Elsevier, which are not offered under a CC BY license and are therefore excluded from this article’s [1] license.

Fig. 5A, the input panel of Fig. 5C, lane 2 of the anti-p65 panel of Fig. 5C, and the TLR4, p-IκBα and IκBα panels in Fig. 5D are similar to material from [5], published in 2012 by Elsevier, which are not offered under a CC BY license and are therefore excluded from this article’s [1] license.

At the time of retraction, the article [1] was republished to note this exclusion in the legends of Figs 2, 4, 5, and 7, and the article’s copyright statement.
